# Hypergolics in Carbon Nanomaterials Synthesis: New Paradigms and Perspectives

**DOI:** 10.3390/molecules25092207

**Published:** 2020-05-08

**Authors:** Nikolaos Chalmpes, Konstantinos Spyrou, Konstantinos C. Vasilopoulos, Athanasios B. Bourlinos, Dimitrios Moschovas, Apostolos Avgeropoulos, Christina Gioti, Michael A. Karakassides, Dimitrios Gournis

**Affiliations:** 1 Department of Materials Science and Engineering, University of Ioannina, 45110 Ioannina, Greece; chalmpesnikos@gmail.com (N.C.); konstantinos.spyrou1@gmail.com (K.S.); kovasil@auth.gr (K.C.V.); dmoschov@cc.uoi.gr (D.M.); aavger@uoi.gr (A.A.); chgioti@cc.uoi.gr (C.G.); mkarakas@uoi.gr (M.A.K.); 2 Physics Department, University of Ioannina, 45110 Ioannina, Greece

**Keywords:** hypergolic reactions, sodium peroxide, carbon nanosheets, fullerols, useful energy

## Abstract

Recently we have highlighted the importance of hypergolic reactions in carbon materials synthesis. In an effort to expand this topic with additional new paradigms, herein we present novel preparations of carbon nanomaterials, such-like carbon nanosheets and fullerols (hydroxylated fullerenes), through spontaneous ignition of coffee-sodium peroxide (Na_2_O_2_) and C_60_-Na_2_O_2_ hypergolic mixtures, respectively. In these cases, coffee and fullerenes played the role of the combustible fuel, whereas sodium peroxide the role of the strong oxidizer (e.g., source of highly concentrated H_2_O_2_). The involved reactions are both thermodynamically and kinetically favoured, thus allowing rapid product formation at ambient conditions. In addition, we provide tips on how to exploit the released energy of such highly exothermic reactions in the generation of useful work.

## 1. Introduction

Carbon plays a central role in material science due to its variety of forms and enchanted properties [1]. Traditionally, carbon synthesis is an energy-consuming process that requires heating of an organic precursor in an oven at elevated temperature for certain periods of time. In this respect, the development of fast, spontaneous and energy-liberating (e.g., exothermic) preparative methods at ambient conditions would be of great value in carbon materials synthesis. Recently, we have introduced hypergolic reactions [2,3,4] as a useful tool in the rapid and spontaneous synthesis of a wide range of carbon nanomaterials at ambient conditions [5,6,7]. It is worth noting, the released energy from such highly exothermic reactions could be further exploited in the generation of useful work (chemical, mechanical, electrical, etc.). In one case, we have shown the formation of carbon nanosheets by the self-ignition of pyrophoric lithium dialkylamides salts in air, with the released energy being utilized for the generation of thermoelectric power [5]. In another case, the spontaneous ignition of an acetylene-chlorine mixture produced highly crystalline graphite at ambient conditions [6]. Lastly, hypergolic mixtures based on nitrile rubber or Girard’s reagent T and fuming HNO_3_ as a strong oxidizer, led to the formation of carbon nanosheets or photoluminescent carbon dot respectively, with the released heat being utilized for the thermal transformation of a triazine precursor into graphitic carbon nitride or of coffee grains into a lightweight carbon absorbent [7].

In an effort to further build upon these results from our group, in the present work we provide additional new paradigms of hypergolic reactions in the service of carbon nanomaterials synthesis. Sodium peroxide Na_2_O_2_ was used as a strong oxidizer [8], the latter being a source of highly concentrated H_2_O_2_ (a reactive oxygen species) upon contact with water. On the other hand, coffee [9,10] and fullerenes C_60_ acted as the carbon source in the corresponding hypergolic mixtures. As far as the carbon materials of interest is concerned here, these included carbon nanosheets [11] and fullerols (hydroxylated fullerenes) [12]. 

In a first paradigm, the ignition of instant coffee grains by Na_2_O_2_ resulted in carbon nanosheets, the latter shown here to be an effective solar energy absorbent. Interestingly, the energy released from the reaction could be drain off to photovoltaics or to the preparation of important magnetic materials, such as magnetic iron oxides from the thermal decomposition of ferric acetate. In a second paradigm, simply crushing C_60_ in the presence of Na_2_O_2_ caused ignition of the mixture with simultaneous formation of fullerols, a well-established fullerene derivative with interesting physico-chemical properties and numerous applications.

## 2. Results and Discussion

### 2.1. Carbon Nanosheets

**Figure 1:**
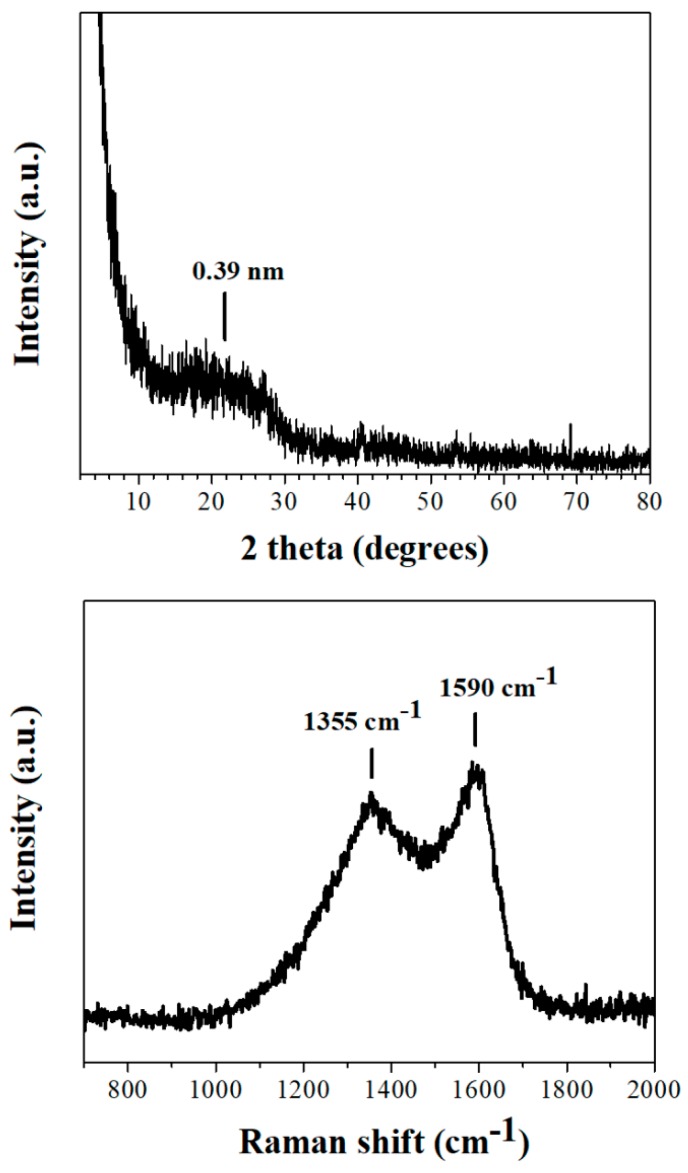
XRD pattern (top) and Raman spectrum (bottom) of the coffee-derived carbon nanosheets.

**Figure 2:**
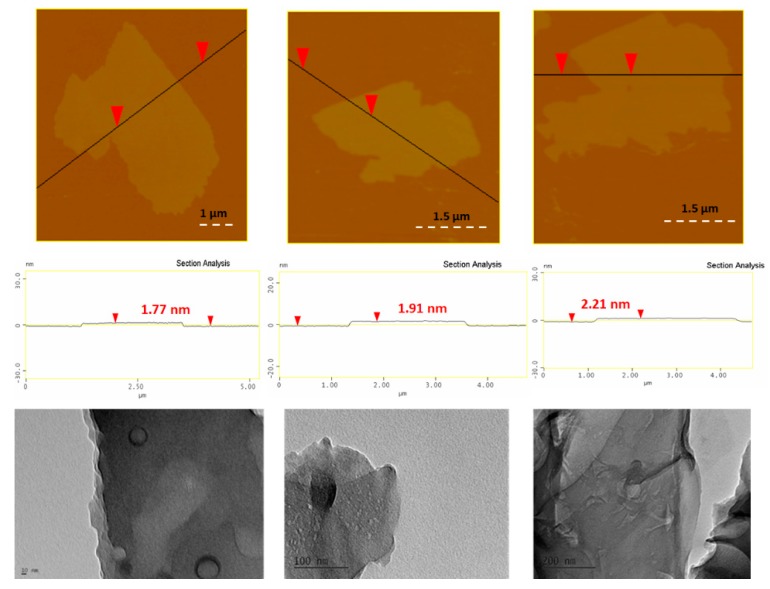
Top: AFM images of cross sectional analysis of selected carbon nanosheets. Bottom: representative TEM images of the sheets.

The XRD pattern of the coffee-derived carbon nanosheets exhibited a very broad reflection centred at d_002_ = 3.9 Å (Figure 1, top), signalling the formation of amorphous carbon [13]. Likewise, Raman spectroscopy gave broad D (1355 cm^−1^) and G (1590 cm^−1^) bands with a relatively intensity ratio of I_D_/I_G_ = 0.9 (Figure 1, bottom), both being typical features of non-crystalline carbon [13]. On the other hand, the XPS spectrum of the nanosheets was identical to those reported in references 5 and 7 for oxidized carbon nanosheets. AFM study of the nanosheets showed the presence of large plates with thickness 1.5–2.5 nm (Figure 2, top). The morphology and size of the sheets were additionally confirmed by TEM microscopy (Figure 2, bottom).

**Figure 3:**
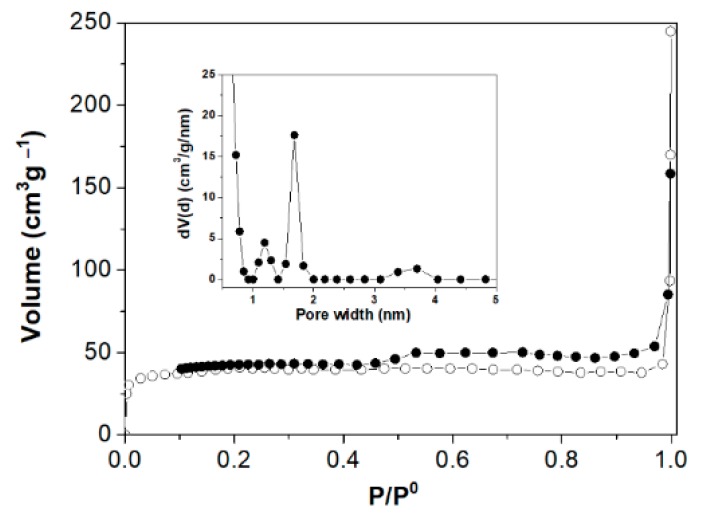
N_2_ adsorption-desorption isotherms of carbon nanosheets. Inset: Quenched Solid Density Functional Theory (QSDFT) pore size distribution.

The N_2_ adsorption-desorption isotherms for the carbon nanosheets is shown in Figure 3. The sample exhibited isotherm of type I according to IUPAC classification, with a H4 type hysteresis loop which is characteristic of slit-shaped pores. The surface area of the sample and the total pore volume were calculated using BET equation (S_BET_) or alternatively, by the “t-plot” and Quenched Solid Density Functional Theory (QSDFT) methods. According to these methods the specific surface area was calculated to be 130 m^2^/g (S_BET_), 164 m^2^/g (S_t_, t-plot) and 179 m^2^/g (QSDFT) respectively, whereas the total pore volume found to be 0.06 cm^3^/g (cumulative at P/P_o_ = 0.97), 0.06 cm^3^/g (t-plot) and 0.085 cm^3^/g (QSDFT). From the pore size distribution PSD (inset) according to the QSDFT model (Figure S1), carbon nanosheets seemed to exhibit micropores with two average sizes 1.2 nm and 1.7 nm and mesoporous 3.7 nm. However, the volume analysis from “t-plot” (Figure S2) showed only the presence of micropores in agreement with classification type of adsorption isotherm. It is obvious that the observed step down in the desorption branch between relative pressures 0.4 to 0.6 (Figure 3), is responsible for the observed mesoporous peak in the calculated PSD. That peak is probably an artefact, caused by the spontaneous evaporation of metastable pore liquid (i.e., cavitation). Besides, H3 or H4 hysteresis is often attributed to the occurrence of pore blocking and percolation phenomena and it is not only associated with the pore condensation [14]. From a practical point of view, the surface area of the nanosheets in combination with the oxygen functionalities present on their surface could make the material useful in adsorption processes (e.g., removal of heavy metals or dyes from water).

**Figure 4:**
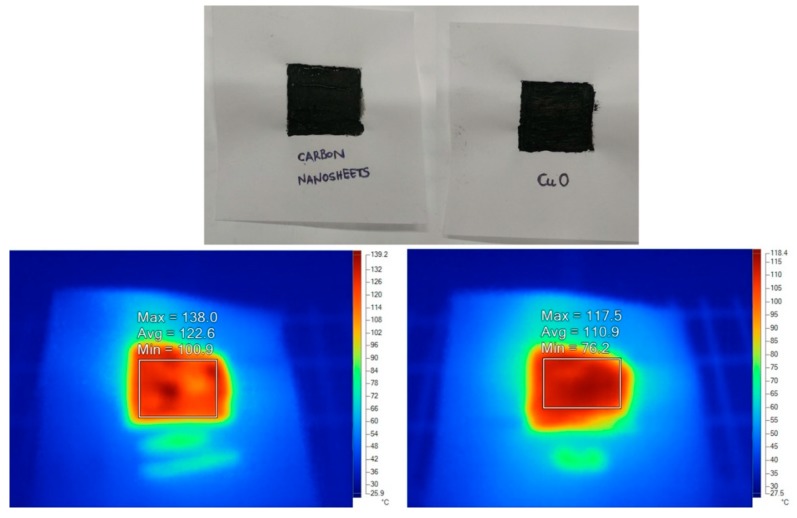
Top: dry carbon nanosheet and CuO paints on paper sheet (black squares). Bottom: the corresponding thermal camera images after 20 s illumination with infrared lamp (left: carbon nanosheets; right: CuO). Both samples developed comparable temperature after illumination under identical conditions, thus demonstrating that the coffee-derived carbon nanosheets are effective solar energy absorbents.

The black solid could be used as a pigment in water glass paints for solar energy harvesting. Water glass refers to an aqueous solution of the inorganic polymer sodium silicate (40%, Aldrich) that can wrap and electrostatically stabilize any dispersed solid. To this aim, carbon nanosheets were simply mechanically mixed with water glass to create the paint. The paint was then spread over a piece of paper with a brush and left to dry at room temperature (see the corresponding black square drawing in Figure 4, top). A similar drawing using an analogous CuO paint was also sketched in a separate piece of paper (Figure 4, top); cupric oxide is a reference black pigment that is often used in solar water heaters [15]. Following, both coatings were illuminated under an infrared lamp at the same distance and for the same time (20 s) prior to scanning with a thermal camera. As it can be seen from the thermal camera images in Figure 4, bottom, carbon nanosheets and cupric oxide developed comparable temperatures (100–120 °C) under identical conditions. Hence, thanks to their flat surface and blackness, the coffee-derived carbon nanosheets could be promising solar energy absorbents [16]. It should be noted that although the CuO film seems homogeneously darker, the nanosheets yield higher temperature rise in places that appear even darker due to a higher mass of deposited material. Thus, the temperature distribution as presented is valid.

**Figure 5:**
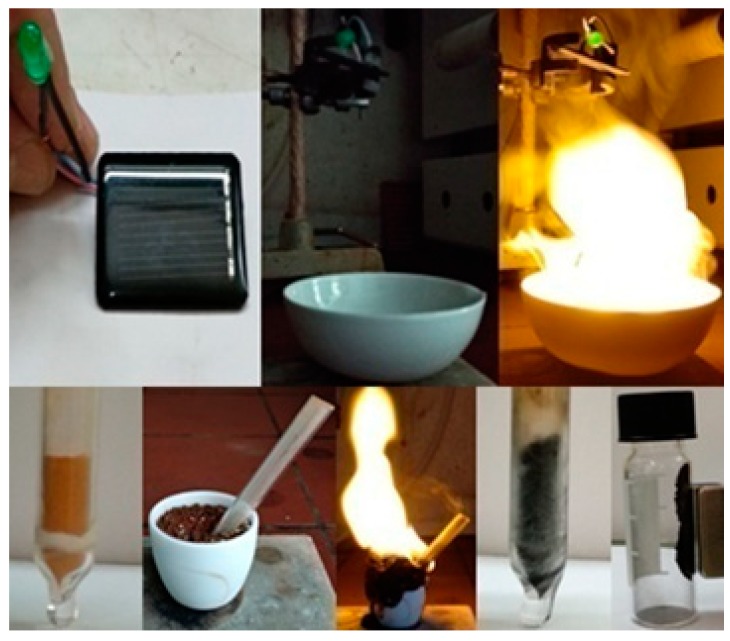
Top: ignition of the coffee-Na_2_O_2_ mixture generates enough thermal radiation and light to turn on a miniature silicon photovoltaic device. Bottom: the ignitable mixture served as a heat source for the in-situ thermal transformation of ferric acetate (orange powder at far-left) into magnetic iron oxide (black solid at far-right). For this purpose, a small quartz tube was charged with the precursor and then merely dipped into the ignition mixture. Notice the release of smoke from the top rim of the tube due to thermal decomposition of ferric acetate.

The heat and light produced from the reaction of the coffee grains with Na_2_O_2_ was utilized in the generation of useful work. In one example, a miniature silicon photovoltaic panel connected with a green LED light was placed above the ignition mixture with the front side facing down the mixture at certain distance (Figure 5, top). The band-gap energy of silicon is 1.1 eV, the latter corresponding to the infrared part of the electromagnetic radiation. Upon ignition, the thermal radiation and light produced from the flame turned on the LED light (Figure 5, top), thus acting as a sort of thermophotovoltaic [17]. In another example, the heat produced from the hypergolic mixture was exploited in the thermal decomposition of ferric acetate into magnetic iron oxide [18,19]. For this purpose, a quartz tube charged with ferric acetate was dipped into an alumina crucible containing the hypergolic mixture (Figure 5, bottom). Ignition of the mixture provided the necessary heat for the thermal decomposition of the precursor inside the tube into magnetic iron oxide (Figure 5, bottom). 

### 2.2. Fullerols

The ignition of coffee by sodium peroxide presented above is considered a classic demonstration experiment in the area of hypergolic reactions that, as shown here, results in carbon nanosheets. Another interesting hypergolic mixture reported for first time is the C_60_-Na_2_O_2_ system (Figure 10). Although several carbon allotropes and their derivatives have been used as booster additives in hypergolic fuels [20], however, it is very seldom to observe spontaneous ignition between elemental carbon and a strong oxidizer upon direct contact. Perhaps a sole example in the literature refers to the activated charcoal-Na_2_O_2_ pair [8] (see also *Bretherick’s Handbook of Reactive Chemical Hazards*). Hence, the C_60_-Na_2_O_2_ system adds another example in the list, thus paving the way for the advancement of novel hypergolics strictly based on carbon. It should be mentioned that similar treatment of other carbon allotropes (e.g., nanodiamonds, carbon nanotubes) with Na_2_O_2_ gave no ignition. Apparently, the small size and strain of fullerenes make them prone to ignition.

**Figure 6:**
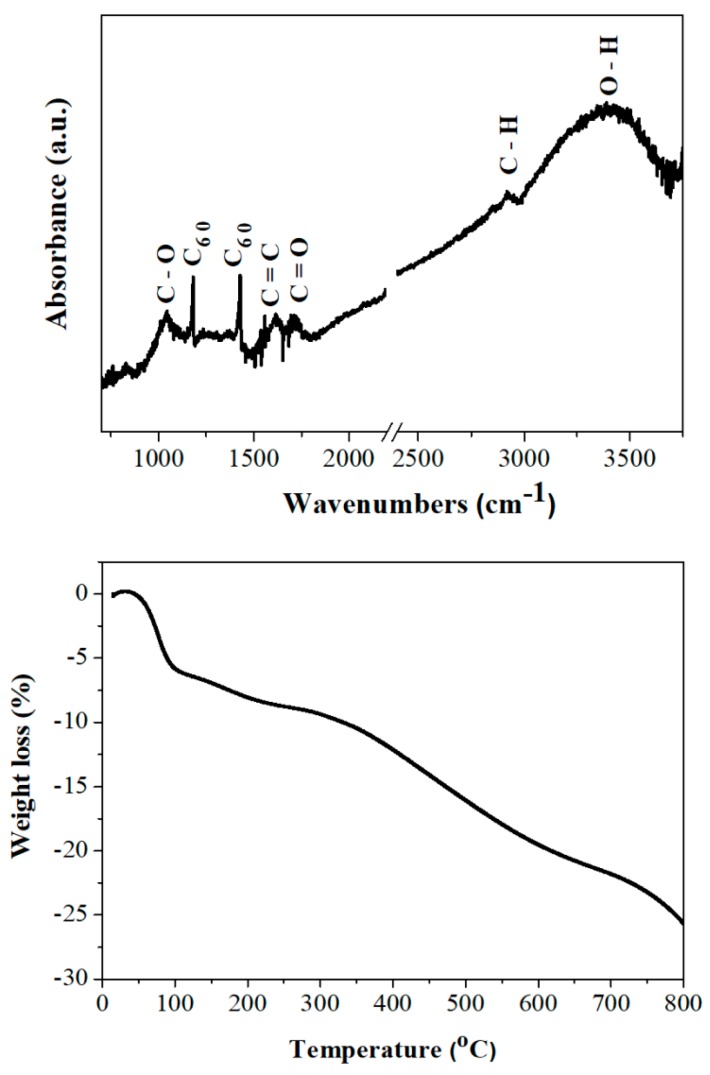
Top: ATR-IR spectrum of the fullerols. Bottom: the corresponding thermogravimetric analysis (TGA) trace under N_2_.

The C_60_-Na_2_O_2_ system is of synthetic value as well, since it can afford fullerols, an important hydroxylated fullerene derivative. In general, the formation of fullerols from C_60_ requires the simultaneous presence of H_2_O_2_ and NaOH [21]. In our case, both chemicals were released in-situ by the peroxide hydrolysis in humid air (e.g., Na_2_O_2_ + 2 H_2_O → H_2_O_2_ + 2 NaOH). The ATR-IR spectrum of the dark brown solid derived from the reaction displayed several broad bands of moderate intensity at 3450 cm^−1^ (O-H), <3000 cm^−1^ (aliphatic C-H), 1717 cm^−1^ (C=O), 1618 cm^−1^ (enol C=C) and 1045 cm^−1^ (C-O) (Figure 6, top). The sharp peaks at 1183 cm^−1^ and 1427 cm^−1^ are ascribed to unreacted C_60_, which cannot be completely removed by further washing (e.g., with toluene). This issue should be cautiously taken into consideration upon scaling-up synthesis. These observations are similar to those reported by Afreen et al. elsewhere for fullerol formation from C_60_ [22]. Especially the O-H, enol C=C and C-O stretchings at 3450 cm^−1^, 1618 cm^−1^ and 1045 cm^−1^ respectively, are indicative of the attachment of -OH groups onto the C_60_ cage. Of particular interest is the appearance of aliphatic C-H and carbonyl C=O bands in the spectrum. The carbonyl group is often ascribed in the literature to the pinacol-pinacolone rearrangement in fullerene 1,2-diols [23]. However, such rearrangement seems rather unlikely for fullerenes since it would require the movement of a fixed skeletal carbon within the cage. In addition, it cannot explain the appearance of C-H aliphatics in the spectrum. Hence, these bands should be better ascribed to the keto-enol tautomerism, which simpler involves the movement of a hydrogen atom from the hydroxyl group of C=C-OH (enol form) to an alpha carbon (e.g., H-C-C=O, keto from). In this way, both C-H and C=O bands can be rationalized in the spectrum. According to TGA under N_2_ (Figure 6, bottom), fullerol exhibited a first weight loss below 150 °C due to the release of hydration water (ca. 8% *w/w*) and another one between 150 and 600 °C due to dehydroxylation of the cluster (ca. 13% *w/w*) [24]. These weight losses agree with the formula C_60_(OH)_7_∙4H_2_O, i.e., low-hydroxylated fullerol [25]. It is worth noting, the latter formula is close to that of C_60_(OH)_8_∙2H_2_O previously reported by Afreen et al. [22]. 

**Figure 7:**
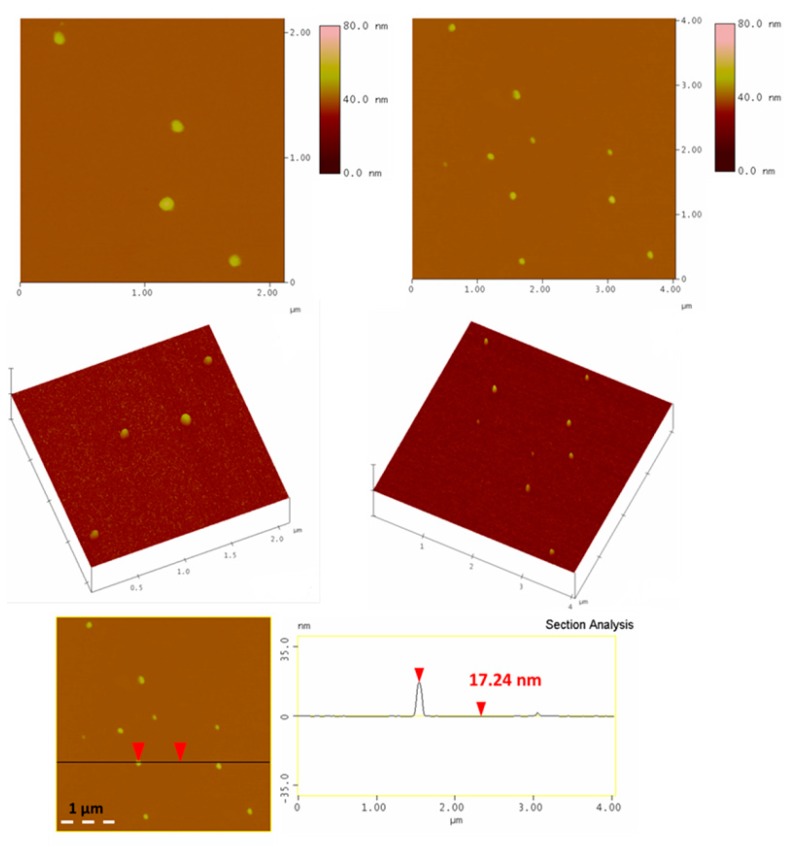
Representative AFM height images (top), 3D morphology (middle) and cross section analysis profile (bottom) of the globular fullerol nanoparticles.

AFM study of the aqueous-dispersed solid revealed the presence of large globular nanoparticles with size in the range of 15–25 nm (Figure 7). Fullerol typically has a size of 1.5 nm; however, it is well-known to form larger aggregates and eventually fullerol nanoparticles (10–60 nm) as a result of strong hydrogen bonding and π–π interactions between adjacent clusters [26]. 

**Figure 8:**
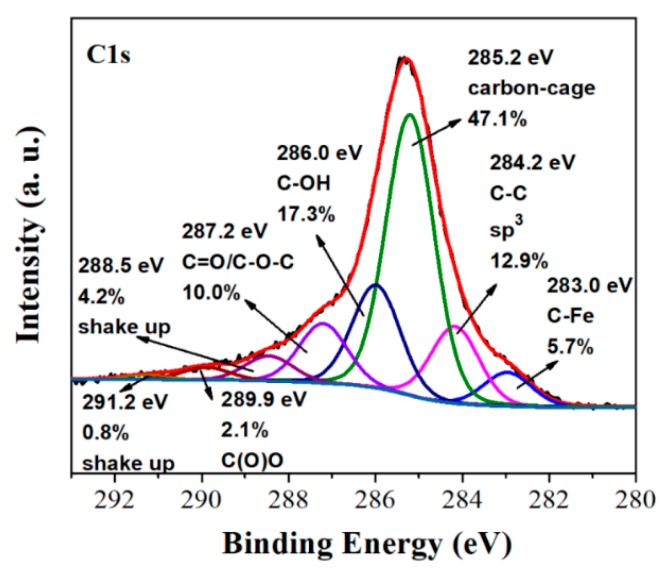
C1s photoelectron spectrum of fullerols.

High resolution C1s XPS gave useful information about the structure of the synthesized fullerols (Figure 8). The dominant peak at 285.2 eV is due to the carbon cage of the fullerols [27]. Additionally, the shake-up features associated with the C_60_-cage atoms were identified at 288.5 eV and 291.2 eV [28,29]. On the other hand, the small fraction of sp^3^ carbons observed at 284.2 eV is consistent with a low degree of functionalization (e.g., low-hydroxylated fullerols). The peak at 286.0 eV is typical of hydroxyl C-OH groups, whereas those at 287.2 and 289.9 eV of C=O/C-O-C/C(O)O species [30]. Lastly, the feature at 283.0 eV is ascribed to iron carbides, the latter resulting from iron contamination of the sample by the metallic container used for reaction.

To sum-up, the ATR-IR, TGA, AFM and XPS data are well-consistent with the formation of low-hydroxylated fullerol, a useful precursor to higher fullerols. 

## 3. Materials and Methods

For safety reasons all experiments were conducted in a fume hood with ceramic tiles bench.

### 3.1. Carbon Nanosheets

**Figure 9:**
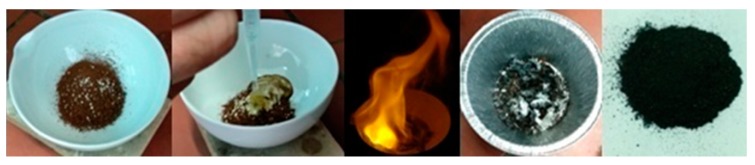
Left-to-right: instant coffee grains were mixed with Na_2_O_2_ beads in a porcelain dish. Subsequent addition of water caused ignition of the mixture towards the formation of a crude carbon residue. After thorough washings, a fine black powder was obtained containing carbon nanosheets.

2 g of instant coffee grains (Nescafe^®^) were mixed with 2 g Na_2_O_2_ beads (93%, Sigma-Aldrich, St. Louis, MO, USA) in a porcelain dish followed by the quick addition of 1 mL water. Ignition started with a short delay (30–45 s after water addition) giving off an intense yellow flame due to the sodium ions present. The reaction led to a crude carbon residue after cooling of the product. Thorough washings with water, dimethylformamide (DMF) and acetone afforded a fine black powder containing carbon nanosheets (yield: 2%). The whole process is visualized in Figure 9. Any minor inorganic contaminants due to the porcelain dish were removed by treating the sample with 48% HF aqueous solution.

### 3.2. Fullerols

**Figure 10:**
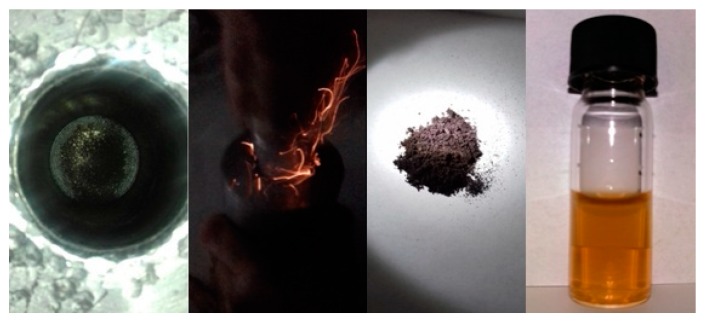
Crushing fullerenes in the presence of Na_2_O_2_ in a stainless steel mortar and pestle caused ignition of the mixture as evidenced by the generation of bright flash. Washing of the product led to a dark brown powder, namely fullerols. Fullerols were soluble in water by mild alkaline treatment and sonication.

150 mg of C_60_ powder (98%, Sigma-Aldrich, St. Louis, MO, USA) were crushed in the presence of 1.7 g Na_2_O_2_ beads (93%, Sigma-Aldrich, St. Louis, MO, USA) using a stainless steel mortar and pestle. Due to rapid oxidation of the carbon clusters by the peroxide, the mixture ignited spontaneously within few seconds. The product washed successively with concentrated HCl aqueous solution (37%) in order to remove excess of Na_2_O_2_ (slow addition), water, and finally acetone. A fine dark brown powder was obtained made up of fullerol clusters (yield: 10%). The clusters were soluble in water by alkaline treatment with NaOH and sonication, due to neutralization of the acidic phenolic protons into phenolate ionic groups [12]. The whole process is visualized in Figure 10. It should be mentioned that the stainless steel mortar and pestle was used in order to crush the very hard Na_2_O_2_ beads. Also, no ignition was observed upon crushing the Na_2_O_2_ beads inside the metallic container in the absence of fullerenes, suggesting that no reaction is taking place between the peroxide and the walls of the metallic container.

At this point we would like to first emphasize that the yields of the above reactions, although low at first glance, are generally considered fair enough in materials science. Second, the selection of a combustible material is rather a trial and error effort at this stage. Several other organic compounds were tested in our lab but failed ignition with sodium peroxide. In many cases though, the reagents reacted exothermically without giving off flame. We speculate that the quantity of mixed reactants play a key role for ignition. Third, the reported hypergolic pairs are unique in terms of matching. For instance, the reaction of coffee grains or C_60_ with fuming HNO_3_ (100%), though exothermic, gave no ignition.

Powder X-ray diffraction (XRD) was performed using background-free Si wafers and Cu Ka radiation from a Bruker Advance D8 diffractometer (Bruker, Billerica, MA, USA). Raman spectra were recorded with a RM 1000 Renishaw micro-Raman system using a laser excitation line at 532 nm (Renishaw, Wotton-under-Edge, England). Attenuated total reflection infrared spectroscopy (ATR-IR) measurements were performed using a Jasco IRT-5000 microscope coupled with a FT/IR-4100 spectrometer (Jasco, Easton, MD, USA). The ZnSe prism of the ATR objective had a 250 μm area in contact with the sample. Background was subtracted and the baseline was corrected for all spectra. Thermogravimetric analysis (TGA) was performed using a Perkin Elmer Pyris Diamond TG/DTA (PerkinElmer, Inc., Waltham, MA, USA). The fullerol sample was heated under N_2_ flow at a rate of 5 °C min^−1^. X-ray photoelectron spectroscopy (XPS) measurements were performed in an ultra-high vacuum at a base pressure of 4 × 10^−10^ mbar with a SPECS GmbH spectrometer equipped with a monochromatic Mg K_α_ source (hv = 1253.6 eV) and a Phoibos-100 hemispherical analyser (Berlin, Germany). Spectral analysis included a Shirley background subtraction and peak separation using Gaussian-Lorentzian functions in a least square fitting program (Winspec) developed at the LISE laboratory, University of Namur, Belgium. The N_2_ adsorption-desorption isotherms were measured at 77 K on a Sorptomatic 1990, ThermoFinnigan porosimeter (Thermo Finnigan LLC, San Jose, CA, USA). Carbon nanosheets were outgassed at 150 °C for 20 h under vacuum before measurements. Specific surface areas were determined with the Brunauer–Emmett–Teller (BET) method. Atomic force microscopy (AFM) images were collected in tapping mode with a Bruker Multimode 3D Nanoscope (Ted Pella Inc., Redding, CA, USA) using a microfabricated silicon cantilever type TAP-300G, with a tip radius of <10 nm and a force constant of approximately 20–75 N m^−1^. The transmission electron microscopy (TEM) study of carbon nanosheets deposited on carbon coated copper grids (CF300-CU-UL, carbon square mesh, CU, 300 mesh from Electron Microscopy Science) was performed using the instrument JEM HR-2100, JEOL Ltd., Tokyo, Japan operated at 200 kV in bright-field mode.

## 4. Conclusions

We have presented new hypergolic reactions towards the synthesis of functional carbon nanomaterials, such-like carbon nanosheets and fullerols. The new systems composed of coffee or fullerene as the combustible fuel and sodium peroxide as the strong oxidizer. In all instances, synthesis was fast, spontaneous and exothermic at ambient conditions, thus enabling not only facile carbon formation but also the generation of useful work (herein photovoltaic or chemical). The coffee-derived carbon nanosheets, due to their flat surface and blackness, seemed to serve as an effective solar energy absorbent, whereas fullerols itself is a well-known fullerene derivative with important properties and uses. Overall, the present work builds upon previous results from our group, showing altogether the wider applicability and generic character of hypergolic reactions in carbon materials synthesis.

## Supplementary Materials

The following are available online at https://www.mdpi.com/1420-3049/25/9/2207/s1, Figure S1: Fitting comparison between experimental data of carbon nanosheets and QSDFT model (N_2_, carbon equilibrium transition kernel at 77 K based on a slit pore model), Figure S2: t-plot for nitrogen adsorbed at 77 K in carbon nanosheets.
