# Supplementary material for: Hypergolics in Carbon Nanomaterials Synthesis: New Paradigms and Perspectives
Source: Molecules. 2020 May 8;25(9):2207. doi: 10.3390/molecules25092207 (PMC7249011; doi:10.3390/molecules25092207)
Supplement: Supplementary file 1 [file molecules-25-02207-s001.zip › molecules-786938-supplementary.pdf]

Supplementary.

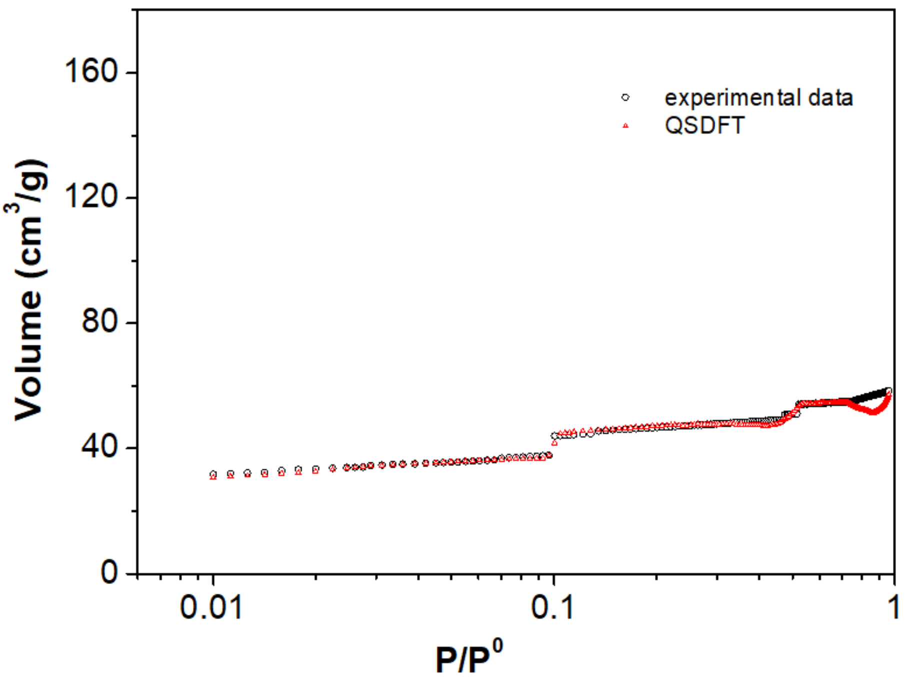

Figure 1. Fitting comparison between experimental data of carbon nanosheets and QSDFT model (N_2, carbon equilibrium transition kernel at 77 K based on a slit pore model).

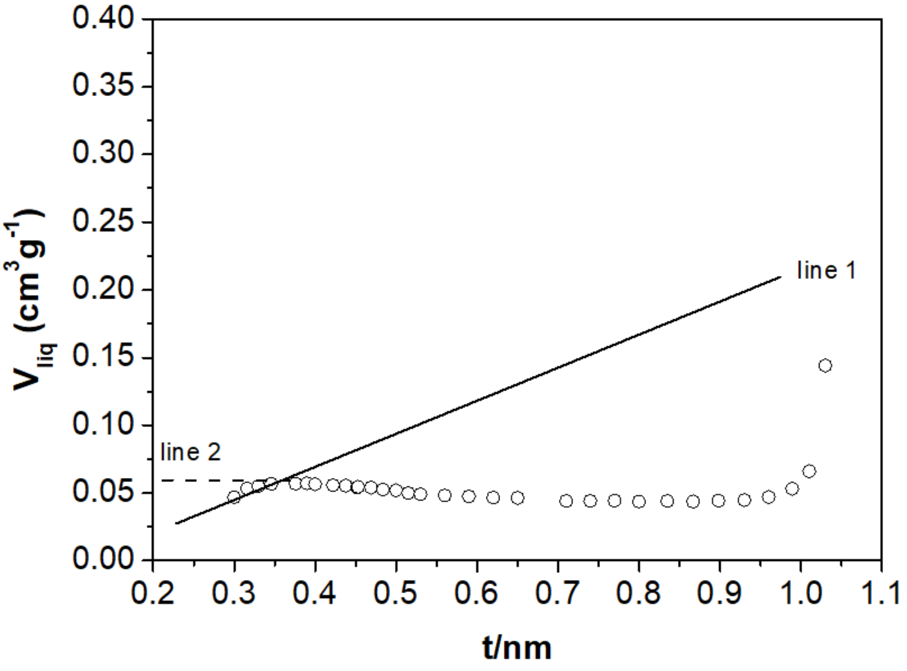

Figure 2. t-plot for nitrogen adsorbed at 77 K in carbon nanosheets. Line 1 has a slope corresponding to the total specific surface area of the sample. The intercept of the line2 with the y-axis provides an estimate of the total porous volume.
